# Supplementary material for: IL-1R signaling drives enteric glia-macrophage interactions in colorectal cancer
Source: Nat Commun. 2024 Jul 19;15:6079. doi: 10.1038/s41467-024-50438-2 (PMC11271635; doi:10.1038/s41467-024-50438-2)
Supplement: Supplementary file 1 — Supplementary Information [file 41467_2024_50438_MOESM1_ESM.pdf]

IL-1R signaling drives enteric glia-macrophage interactions in colorectal cancer

Supplementary Table 1. FACS Antibodies

Anti	Conjugate	Company	Cat. No.	Clone	Dilution
ARG1	Pe-Cy7	eBioscience	25-3697-82	A1efF5	1:100
C1Q	FITC	Tebubio	7501F	RmC7H8	1:50
CD11b	BUV395	BD Horizon	563553	M1/70	1:1000
CD11b	PE-Cy7	BD Pharmingen	552850	M1/70	1:400
CD19	PE-Cy5	eBioscience	15-0193-82	eBio1D3	1:400
CD3	Alexa Fluor 700	BioLegend	100215	17A2	1:200
CD31	BV421	BioLegend	102423	390	1:100
CD326	PE-Cy7	BioLegend	118215	G8.8	1:200
CD3e	eFluor 450	eBioscience	48-0032-82	17A2	1:100
CD4	BV605	BioLegend	100548	RM4-5	1:400
CD44	AF700	BioLegend	103026	IM7	1:200
CD45	BUV805	BD OptiBuild	748370	30-F11	1:1000
CD45	APC-eFluor 780	eBioscience	47-0451-82	30-F11	1:250
CD64	BV711	BioLegend	139311	X54-5/7.1	1:200
CD8a	APC-Cy7	eBioscience	25-5773-82	FJK-16s	1:200
FOXP3	PE-Cy7	eBioscience	12-5773-82	FJK16S	1:50
GP38 (PDPN)	Alexa Fluor 488	BioLegend	127405	8.1.1	1:100
IL-1α	PE	BioLegend	503203	ALF-161	1:50
IL-1β (Pro-form)	APC	eBioscience	17-7114-80	NJTEN3	1:50
Live Dead	eFluor 506	eBioscience	65-0866-14		1:400
Live Dead	7-AAD	BD Pharmingen	51-68981E		1:100
Live Dead	Hoechst	Invitrogen	H3569		1:1000
Ly6C	BV421	BioLegend	128043	HK1.4	1:500
Ly6C	BV650	BioLegend	128049	HK1.4	1:200
Ly6C	FITC	BD Pharmingen	553104	AL-21	1:300
Ly6G	BUV563	BD Horizon	612921	IA8	1:250

Ly6G	APC	BD Pharmingen	560599	1A8	1:300
MHCII	APC-eFluor 780	eBioscience	47-5321-82	M5/114.15.2	1:400
MHCII	BV510	BioLegend	107636	M5/114.15.2	1:500
SiglecF	eFluor 660	eBioscience	50-1702-80	1RNM44N	1:200
SPP1	PE	R&D systems	IC808P		1:25

Supplementary Table 2. Primer Sequences

	Forward primer	Reverse primer
Arg1	CAGAAGAATGGAAGAGTCAG	CAGATATGCAGGGAGTCACC
C1qa	ATCCAGTTTGATCGGACCAC	CATCTTCAGCCACTGTCCATA
Ccl2	CAGGTGTCCCAAAGAAGCTGTA	CATTTGGTTCCGATCCAGG
Ifng	TCAAGTGGCATAGATGTGGAAGAA	TGGCTCTGCAGGATTTTCATG
Il1a	GAGAGCCGGGTGACAGTATC	ACTTCTGCCTGACGAGCTTC
Il1b	TTGACGGACCCCAAAAGATG	AGGACAGCCCAGGTCAAAG
Il6	CCATAGCTACCTGGAGTACATG	TGGAAATTGGGGTAGGAAGGAC
Lcn2	CGGAGCGATCAGTTCCGGG	GCCCTGGTCCTGGTCCCTGA
Rpl32	AAGCGAAACTGGCGGAAAC	TAACCGATGTTGGGCATCAG
S100b	GGTTGCCCTCATTGATGTCTTCC	CTTCCTGCTCCTTGATTTCCTCCA
Sox10	GACACTAGGCAAGCTCTGGAGGTT	CCTCCTTGCTCGGCTTCCC
Spp1	AGCAAGAAACTCTTCCAAGCAA	GTGAGATTCGTCAGATTCATCCG
Timp1	AGACAGCCTTCTGCAACT	CAGCCTTGAATCCTTTTAGCATC
Tnfa	CTATGGCCCAGACCCTCACACTC	GCTGGCACCACTAGTTGGTTGTCTT

Supplementary Table 3

	Gene signature
Enteric glial cells (manually curated from literature [1–3])	S100B, SOX10, PLP1, GFAP, CRYAB, CLU, FXYD1, ALDH1A1, PMP22, CDH19, SCN7A, PRNP
SPP1^{+} TAMs [4]	SPP1, PCSK5, SLC11A1, VCAN, SLC25A37, FLNA, UPP1, BCL6, AQP9, TIMP1, VEGFA, ADM, MARCO, FN1, IL1RN

SUPPLEMENTARY FIGURES

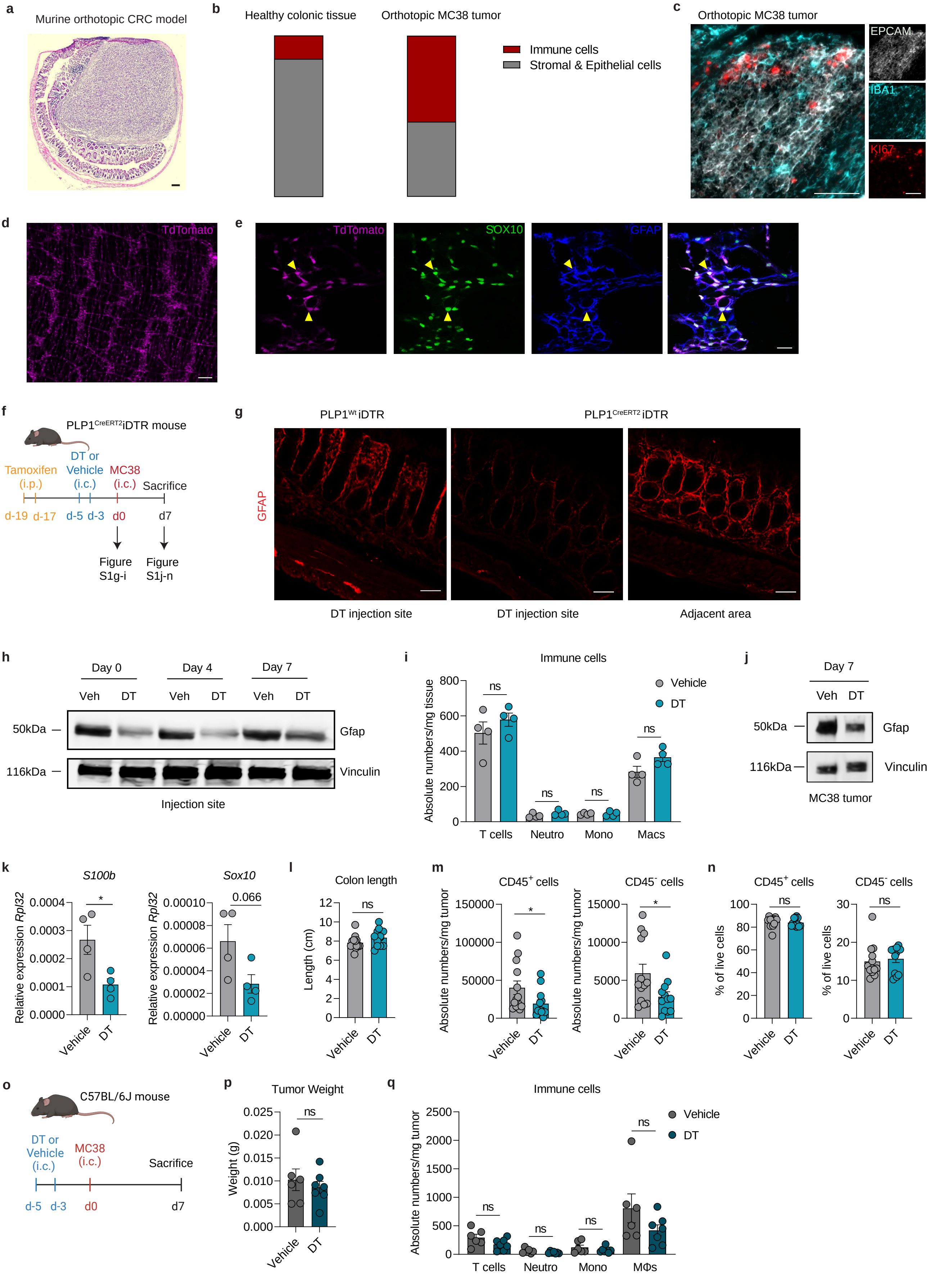

a
Murine orthotopic CRC model
b
Healthy colonic tissue
Orthotopic MC38 tumor
Immune cells
Stromal & Epithelial cells
c
Orthotopic MC38 tumor
EPCAM
IBA1
KI67
d
TdTomato
e
TdTomato
SOX10
GFAP
f
PLP1CreERT2iDTR mouse
Tamoxifen (i.p.)
DT or Vehicle (i.c.)
MC38 (i.c.)
Sacrifice
d-19 d-17 d-5 d-3 d0 d7
Figure S1g-i
Figure S1j-n
g
PLP1Wt iDTR
PLP1CreERT2 iDTR
GFAP
DT injection site
DT injection site
Adjacent area
h
Day 0
Day 4
Day 7
Veh DT Veh DT Veh DT
50kDa
116kDa
Gfap
Vinculin
Injection site
i
Immune cells
Absolute numbers/mg tissue
Vehicle
DT
ns
T cells
Neutro
Mono
Macs
j
Day 7
Veh DT
50kDa
116kDa
Gfap
Vinculin
MC38 tumor
k
S100b
Sox10
Relative expression Rpl32
*
0.066
Vehicle
DT
l
Colon length
Length (cm)
ns
m
CD45+ cells
CD45- cells
Absolute numbers/mg tumor
*
n
CD45+ cells
CD45- cells
% of live cells
ns
o
C57BL/6J mouse
DT or Vehicle (i.c.)
MC38 (i.c.)
Sacrifice
d-5 d-3 d0 d7
p
Tumor Weight
Weight (g)
ns
q
Immune cells
Absolute numbers/mg tumor
Vehicle
DT
T cells
Neutro
Mono
MΦs

Supplementary Figure 1. MC38 orthotopic mouse model, as a reliable model to study EGC-induced immune regulation in colorectal cancer.

a-c MC38 orthotopic CRC tumor, 21 days after injection. Hematoxylin and eosin image, scale bar 500 µm (*n = 13*) (**a**), proportional representation of (non)-immune cells in healthy colon and CRC tissues (*n = 6*) (**b**) and immunofluorescent images showing EPCAM (grey), IBA1 (cyan) and KI67 (red), scale bar 50 µm *(n = 3)* (**c**). **d-e** Healthy muscularis tissue in PLP1^{CreERT2}Ai14$^{fl/fl}$ mice showing tdTomato (magenta), SOX10 (green) and GFAP (blue), scale bar 100 µm (**d**) and 25 µm (**e**) (*n = 2*). **f-n** After tamoxifen treatment, PLP1^{CreERT2}iDTR mice were intracolonically (i.c.) injected at d-5 and d-3 with 40 ng Diphtheria toxin (DT) or saline (Vehicle). On d0 both groups were i.c. injected with MC38 cells. Schematic representation (**f**). GFAP (red) staining in the colon at d0 of PLP1^{Wt}iDTR and PLP1^{CreERT2}iDTR mice, scale bar 50 µm (*n = 2* Wt, *n = 5* CreERT2) (**g**). Western blot analysis of GFAP and Vinculin at the injection site on d0, d4, and d7 (**h**). Absolute numbers of immune cells/mg (*n = 4* mice) (**i**) of colon tissue at the site of injection on d0*.* Western blot analysis of GFAP and Vinculin in tumor tissue on d7 (**j**). Relative mRNA levels for *S100b* and *Sox10* normalized to *Rpl32* (*n = 4* Vehicle, *n = 5* DT) (**k**). Colon length (*n = 9* Vehicle, *n = 7* DT) (**l**). Absolute numbers/mg tumor (**m**) and % of live cells (**n**) for immune (CD45^{+}) and non-immune (CD45^{-}) cells (*n = 13* Vehicle, *n = 12* DT) at d7. **o-q** C57BL/6J mice were i.c. injected at d-5 and d-3 with 40 ng DT or saline. At d0, MC38 cells were i.c. injected in both groups. Schematic representation of tumor model (**o**) with quantitative comparison of tumor weight (**p**) and absolute tumor-infiltrating immune cell numbers/mg tumor at d7 (**q**) *(n = 6* Vehicle*, n = 7* DT). Data show mean ± SEM (i, k-n, p-q). Statistical analysis: One-way ANOVA test with correction for multiple comparisons (i,q), unpaired t-test (k) and unpaired Mann-Whitney test (l-n, p). *p < 0.05, ns not significant. Source data are provided as Source Data file.

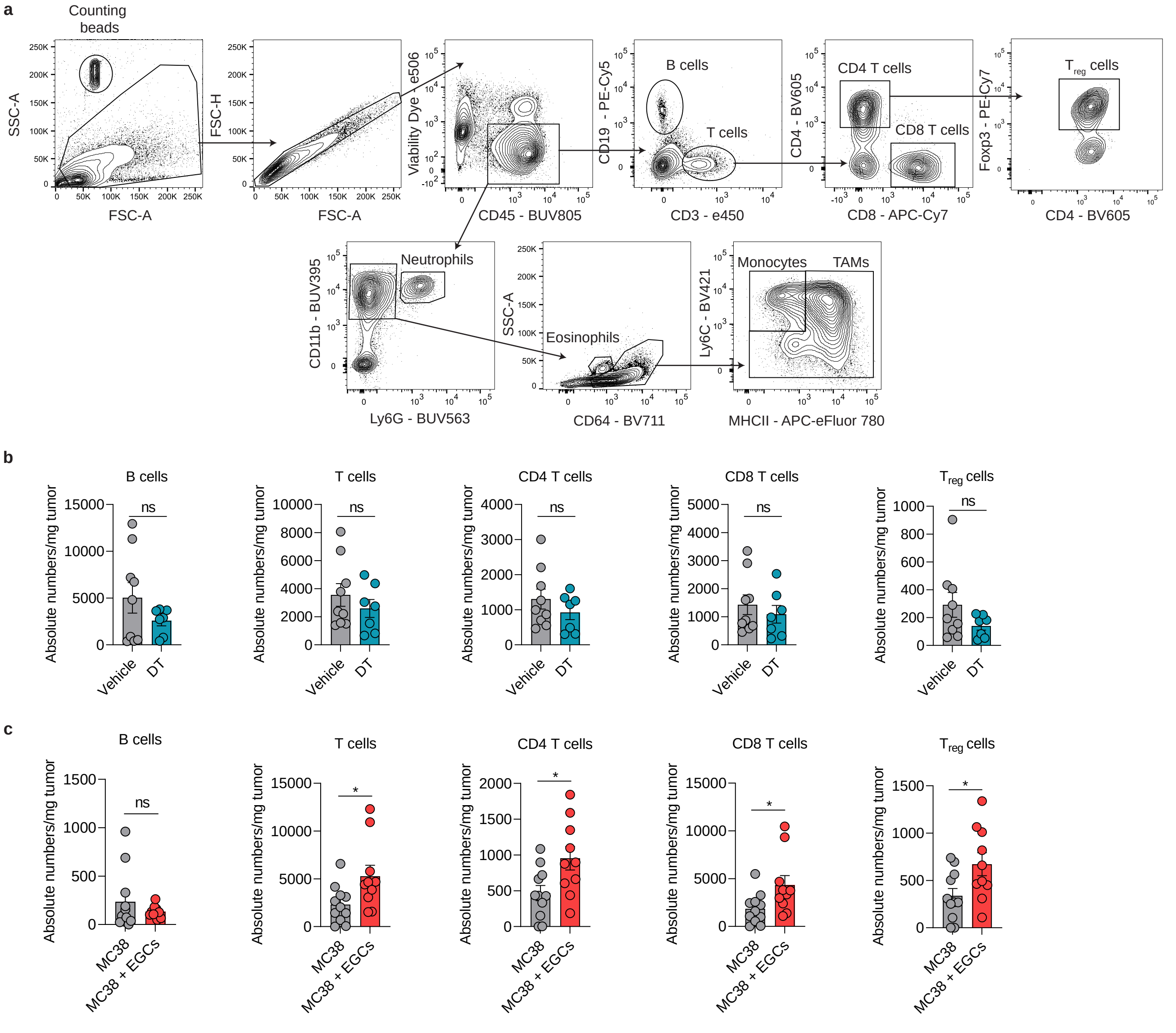
a
Counting beads
SSC-A
FSC-A
FSC-H
Viability Dye - e506
CD45 - BUV805
CD19 - PE-Cy5
CD3 - e450
B cells
T cells
CD4 - BV605
CD8 - APC-Cy7
CD4 T cells
CD8 T cells
Foxp3 - PE-Cy7
Treg cells
CD11b - BUV395
Ly6G - BUV563
Neutrophils
CD64 - BV711
Eosinophils
Ly6C - BV421
MHCII - APC-eFluor 780
Monocytes
TAMs
b
B cells
T cells
CD4 T cells
CD8 T cells
Treg cells
Absolute numbers/mg tumor
ns
Vehicle
DT
c
B cells
T cells
CD4 T cells
CD8 T cells
Treg cells
Absolute numbers/mg tumor
ns
*
MC38
MC38 + EGCs

Supplementary Figure 2. EGCs dictate the immune populations in the TME.

a FACS gating strategy to identify the lymphoid (up) and myeloid (down) populations in the tumor microenvironment. **b** PLP1^{CreERT2}iDTR mice were intracolonically (i.c.) injected at d-5 and d-3 with 40 ng Diphtheria toxin (DT) or saline (Vehicle). On d0 both groups were i.c. injected with MC38 cells and on d7 the tumor-infiltrating lymphoid immune cells were assessed by flow cytometry. Data of immune cells are presented as absolute numbers per mg of tumor tissue (*n = 9* Vehicle, *n = 7* DT). **c** WT C57BL/6J mice were i.c. injected with MC38 cells with or without embryonic neurosphere-derived EGCs (1:1 ratio). The tumor-infiltrating lymphoid immune cells were assessed by flow cytometry on d21. Data are presented as absolute numbers per mg of tumor tissue (*n = 11* MC38, *n = 10* MC38 + EGCs). Data are represented as mean ± SEM (b,c). Statistical analysis: unpaired Mann-Whitney (b,c) *$p < 0.05$, ns not significant. Source data are provided as a Source Data file.

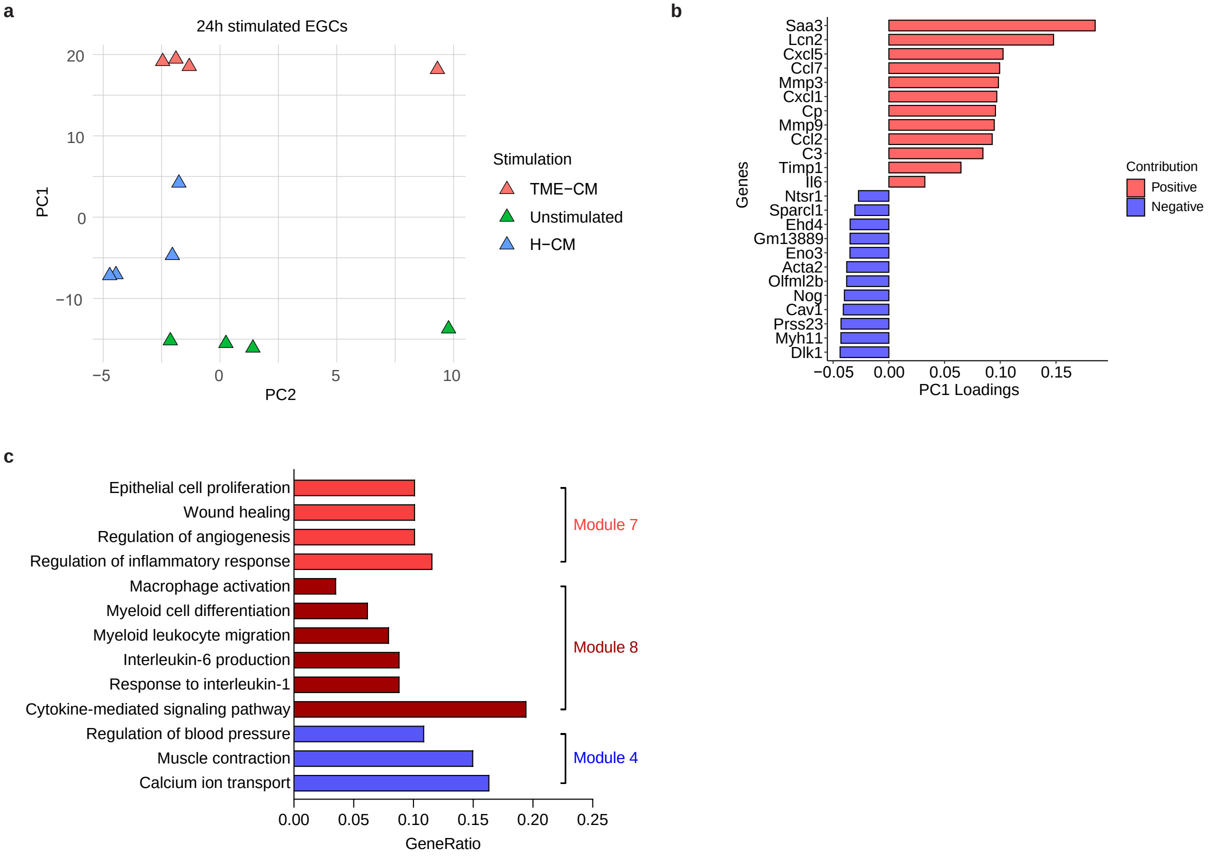
a
24h stimulated EGCs
PC1
20
10
0
−10
−5
0
5
10
PC2
Stimulation
TME−CM
Unstimulated
H−CM
b
Genes
Saa3
Lcn2
Cxcl5
Ccl7
Mmp3
Cxcl1
Cp
Mmp9
Ccl2
C3
Timp1
Il6
Ntsr1
Sparcl1
Ehd4
Gm13889
Eno3
Acta2
Olfml2b
Nog
Cav1
Prss23
Myh11
Dlk1
−0.05
0.00
0.05
0.10
0.15
PC1 Loadings
Contribution
Positive
Negative
c
Epithelial cell proliferation
Wound healing
Regulation of angiogenesis
Regulation of inflammatory response
Macrophage activation
Myeloid cell differentiation
Myeloid leukocyte migration
Interleukin-6 production
Response to interleukin-1
Cytokine-mediated signaling pathway
Regulation of blood pressure
Muscle contraction
Calcium ion transport
Module 7
Module 8
Module 4
0.00
0.05
0.10
0.15
0.20
0.25
GeneRatio

Supplementary Figure 3. Signature genes and functions associated with CRC EGCs.

Transcriptome analysis of *in vitro* primary embryonic neurosphere-derived EGCs alone or stimulated with healthy conditioned medium (H-CM) or tumor microenvironment conditioned medium (TME-CM) at different time points (6h, 12h, and 24h, $n = 4$). **a** Principal component analysis (PCA) plot of EGCs gene signatures of 24h stimulated samples only. Each dot represents an individual sample. **b** Barplot showing genes with positive and negative contributions towards PC1 of Supplementary Fig. 3a. **c** Barplot showing the Gene set enrichment analysis for the transcriptional modules 4, 7 and 8 identified by weighted gene correlation network analysis (WGCNA) (all terms have adjusted p value <0.05). Source data are provided as a Source Data file.

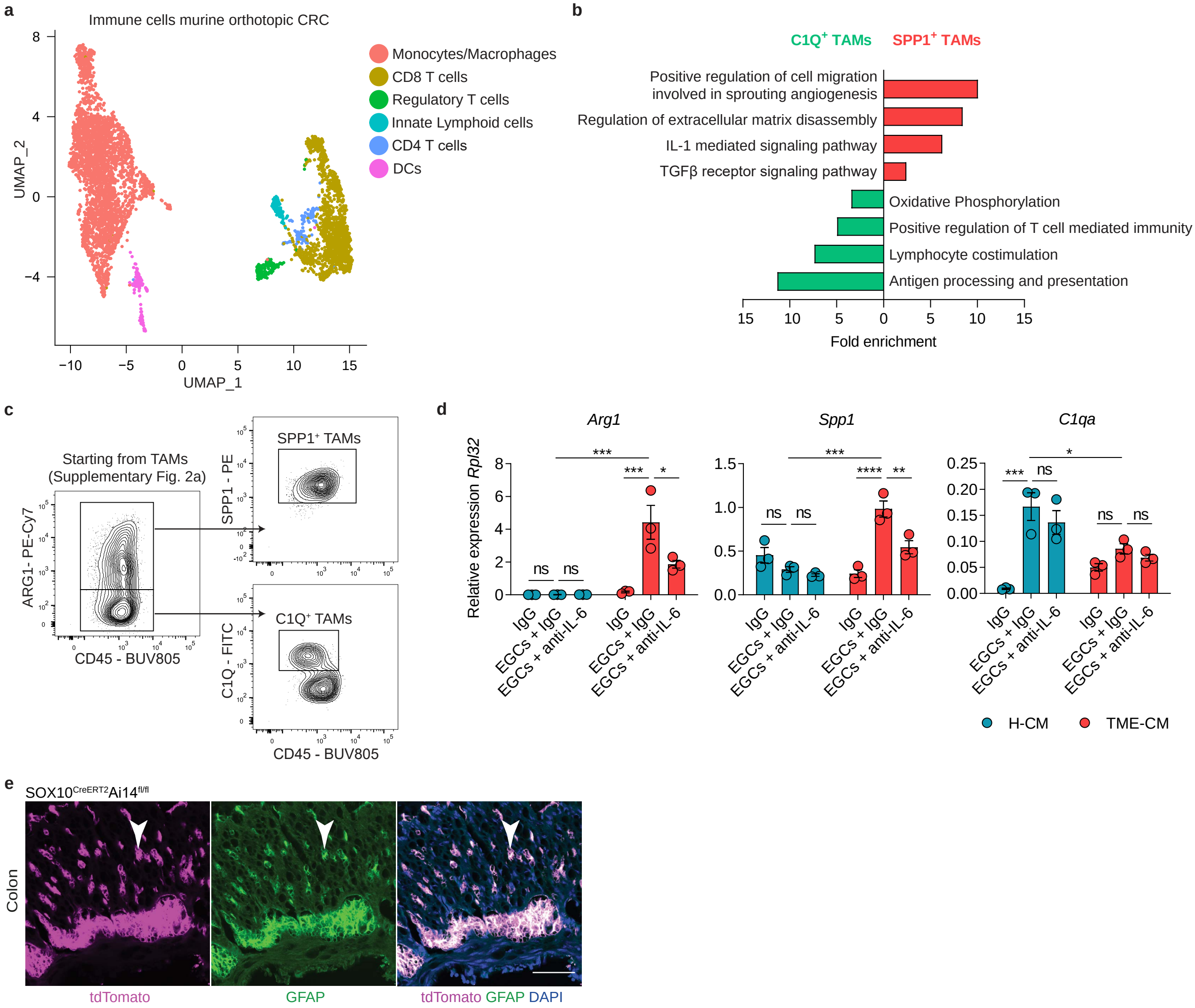
a
Immune cells murine orthotopic CRC
Monocytes/Macrophages
CD8 T cells
Regulatory T cells
Innate Lymphoid cells
CD4 T cells
DCs
UMAP_1
UMAP_2
b
C1Q+ TAMs
SPP1+ TAMs
Positive regulation of cell migration involved in sprouting angiogenesis
Regulation of extracellular matrix disassembly
IL-1 mediated signaling pathway
TGFβ receptor signaling pathway
Oxidative Phosphorylation
Positive regulation of T cell mediated immunity
Lymphocyte costimulation
Antigen processing and presentation
Fold enrichment
c
Starting from TAMs (Supplementary Fig. 2a)
ARG1- PE-Cy7
CD45 - BUV805
SPP1+ TAMs
SPP1 - PE
C1Q+ TAMs
C1Q - FITC
d
Arg1
Spp1
C1qa
Relative expression Rpl32
IgG
EGCs + IgG
EGCs + anti-IL-6
H-CM
TME-CM
e
SOX10CreERT2Ai14fl/fl
Colon
tdTomato
GFAP
tdTomato GFAP DAPI

Supplementary Figure 4. Dichotomy of functional phenotypes of TAMs in murine orthotopic CRC

a UMAP of scRNA-seq data from tumor-infiltrating immune cells in WT C57BL/6J mice bearing orthotopic colon tumors, d21 after tumor induction *(n = 3).* **b** Gene ontology biological process (GOBP) analysis showing the differential pathways enriched in SPP1^{+} TAMs versus C1Q^{+} TAMs, data extracted from scRNAseq murine orthotopic CRC dataset. **c** SPP1^{+} and C1Q^{+} TAMs flow cytometry gating strategy. **d** Supernatant of healthy (H) conditioned medium (-CM), tumor microenvironment (TME)-CM, H EGCs-CM, and TME EGCs-CM was incubated with either IgG or anti-IL-6 (both 5 µg/mL) along with Dynabeads™ Protein G followed by removal of the protein-antibody-bead complex. *In vitro* murine bone marrow-derived monocytes were cultured for 48h with the different supernatants. Relative mRNA levels for *Arg1, Spp1,* and *C1qa* normalized to the housekeeping gene *Rpl32* in monocytes after stimuli (*n = 3*). **e** Representative image showing tdTomato (magenta), GFAP (green), and DAPI (blue) in colon tissue section (scale bar 50 µm) of EGC reporter SOX10^{CreERT2}Ai14$^{fl/fl}$ mice (*n = 3*). Data are represented as mean ± SEM (d). Statistical analysis: two-way ANOVA with correction for multiple comparisons (d). * $p < 0.05$, *** $p < 0.0005$, **** $p < 0.00005$, ns not significant. Source data are provided as a Source Data file.

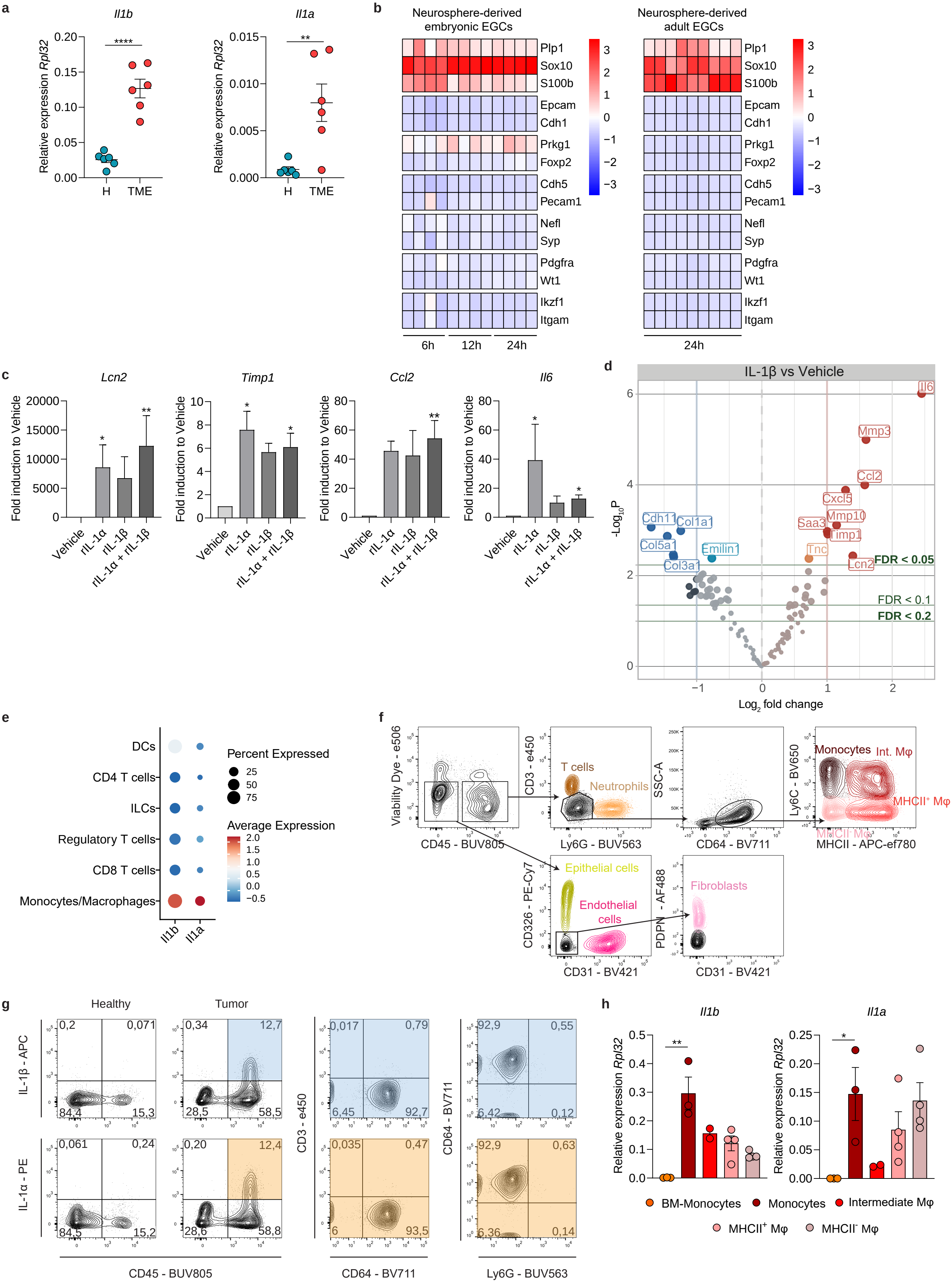
a
Il1b
Il1a
Relative expression Rpl32
****
**
H
TME
0.00
0.05
0.10
0.15
0.20
0.000
0.005
0.010
0.015
b
Neurosphere-derived embryonic EGCs
Neurosphere-derived adult EGCs
Plp1
Sox10
S100b
Epcam
Cdh1
Prkg1
Foxp2
Cdh5
Pecam1
Nefl
Syp
Pdgfra
Wt1
Ikzf1
Itgam
6h
12h
24h
3
2
1
0
−1
−2
−3
c
Lcn2
Timp1
Ccl2
Il6
Fold induction to Vehicle
Vehicle
rIL-1α
rIL-1β
rIL-1α + rIL-1β
*
**
d
IL-1β vs Vehicle
-Log10P
Log2 fold change
Il6
Mmp3
Ccl2
Cxcl5
Mmp10
Saa3
Timp1
Tnc
Lcn2
Cdh11
Col1a1
Col5a1
Emilin1
Col3a1
FDR < 0.05
FDR < 0.1
FDR < 0.2
e
DCs
CD4 T cells
ILCs
Regulatory T cells
CD8 T cells
Monocytes/Macrophages
Il1b
Il1a
Percent Expressed
25
50
75
Average Expression
2.0
1.5
1.0
0.5
0.0
−0.5
f
Viability Dye - e506
CD45 - BUV805
CD3 - e450
Ly6G - BUV563
T cells
Neutrophils
SSC-A
CD64 - BV711
Ly6C - BV650
MHCII - APC-ef780
Monocytes
Int. Mφ
MHCII+ Mφ
MHCII- Mφ
CD326 - PE-Cy7
CD31 - BV421
Epithelial cells
Endothelial cells
PDPN - AF488
Fibroblasts
g
Healthy
Tumor
IL-1β - APC
IL-1α - PE
CD45 - BUV805
CD3 - e450
CD64 - BV711
CD64 - BV711
Ly6G - BUV563
0,2
0,071
84,4
15,3
0,34
12,7
28,5
58,5
0,061
0,24
84,5
15,2
0,20
12,4
28,6
58,8
0,017
0,79
6,45
92,7
0,035
0,47
6
93,5
92,9
0,55
6,42
0,12
92,9
0,63
6,36
0,14
h
Il1b
Il1a
Relative expression Rpl32
**
*
BM-Monocytes
Monocytes
Intermediate Mφ
MHCII+ Mφ
MHCII- Mφ

Supplementary Figure 5. TME-derived IL-1 induces the CRC EGCs signature *in vitro*

a Relative mRNA levels for *Il1b* and *Il1a* normalized to the housekeeping gene *Rpl32,* in single-cell suspensions of murine healthy colon (H) or orthotopic tumor microenvironment (TME) tissue *(n = 6)*. **b** Heatmap illustrating the expression levels of marker genes associated with different cell types in unstimulated samples collected at indicated time points from bulk RNA-seq data of embryonic neurosphere-derived EGCs (*n = 4*) and adult neurosphere-derived EGCs (*n = 9*). **c** Relative mRNA levels for *Lcn2, Timp1, Ccl2*, and *Il6* in primary embryonic neurosphere-derived EGCs stimulated for 24h with or without recombinant (r) IL-1α, IL-1β (each 10 ng/mL) or both (*n = 6* vehicle, *n = 3* rIL-1α and rIL-1β, *n = 6* rIL-1α + rIL-1β). **d** Volcano plot of differentially expressed proteins between primary adult neurosphere-derived EGCs treated for 24h with rIL-1β (10 ng/ml) or vehicle. Protein concentration in the supernatants was determined by liquid chromatography/mass spectrometry (*n = 4*). **e** Dot plot showing expression of *Il1b* and *Il1a* in the tumor-infiltrating immune cell clusters identified by scRNA-seq analysis of orthotopic colon tumors (*n = 3*). **f** FACS gating strategy to identify immune and stromal populations in the TME (Int. MΦ, intermediate Macrophages). **g** Contour plots representing IL-1β and IL-1α expression in healthy colon and orthotopic tumors based on CD45 (left), CD3 and CD64 (middle) and CD64 and Ly6G expression (right). **h** Relative mRNA levels for *Il1b* and *Il1a* normalized to the housekeeping gene *Rpl32* in tumor-infiltrating monocytes, Int. MΦ, $MHCII^{+}$ MΦ and $MHCII^{-}$ MΦ and bone marrow (BM)-derived monocytes from mice bearing orthotopic colon tumors (*n = 3* BM-derived monocytes and tumor-infiltrating monocytes, *n = 2* intermediate MΦ and *n = 4* $MHCII^{+}$ MΦ and $MHCII^{-}$ MΦ). Data are represented as mean ± SEM (a, c, h). Statistical analysis: unpaired t-test (a), Kruskal-Wallis test with correction for multiple comparisons, compared to Vehicle (c) or one-way ANOVA with correction for multiple comparisons (h) *p < 0.05, ** p <0.005, **** p <0.00005, ns not significant. Source data are provided as a Source Data file.

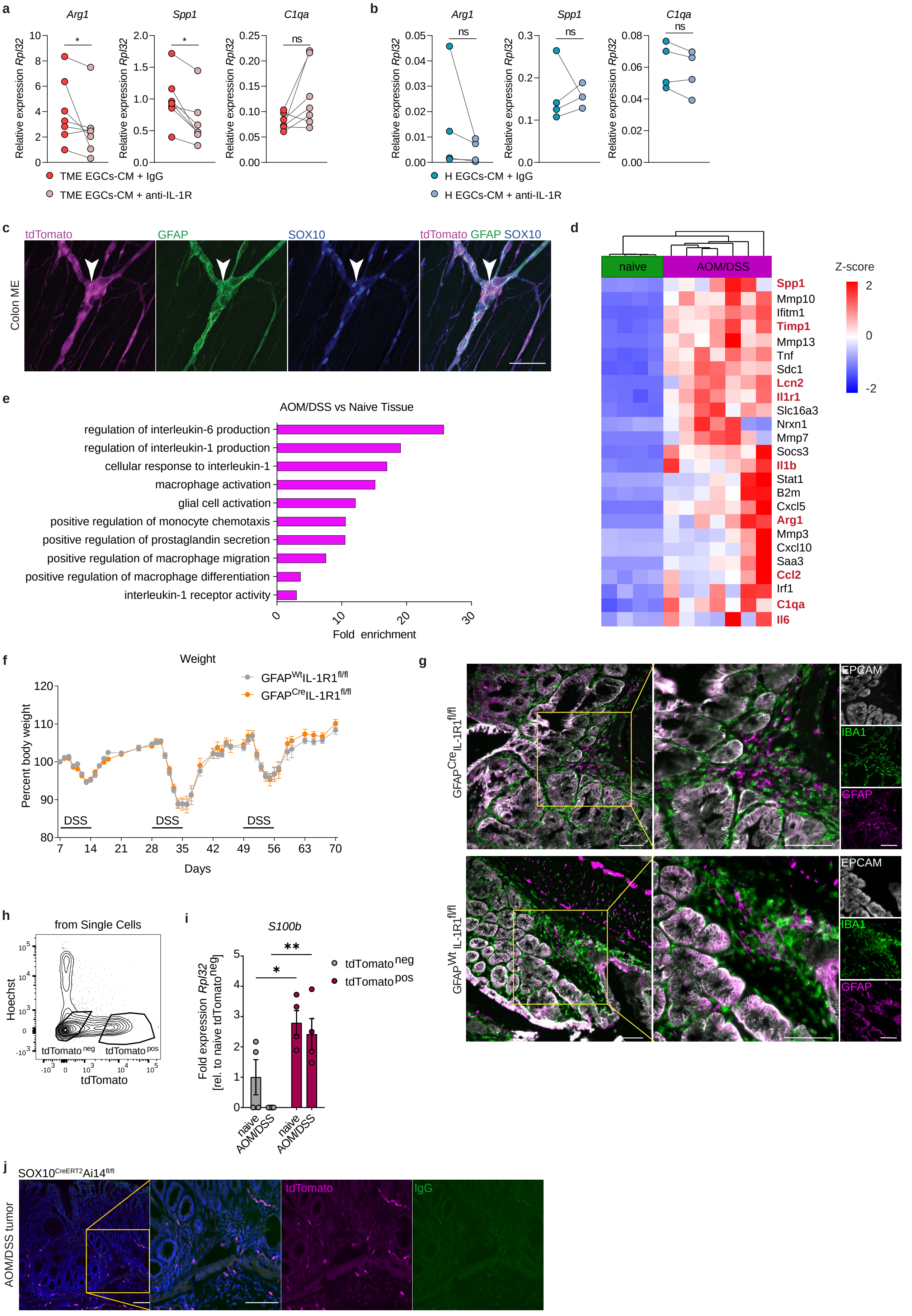

a
Arg1
Spp1
C1qa
Relative expression Rpl32
TME EGCs-CM + IgG
TME EGCs-CM + anti-IL-1R
b
H EGCs-CM + IgG
H EGCs-CM + anti-IL-1R
ns
*
c
tdTomato
GFAP
SOX10
Colon ME
d
naive
AOM/DSS
Z-score
Spp1
Mmp10
Ifitm1
Timp1
Mmp13
Tnf
Sdc1
Lcn2
Il1r1
Slc16a3
Nrxn1
Mmp7
Socs3
Il1b
Stat1
B2m
Cxcl5
Arg1
Mmp3
Cxcl10
Saa3
Ccl2
Irf1
C1qa
Il6
e
AOM/DSS vs Naive Tissue
regulation of interleukin-6 production
regulation of interleukin-1 production
cellular response to interleukin-1
macrophage activation
glial cell activation
positive regulation of monocyte chemotaxis
positive regulation of prostaglandin secretion
positive regulation of macrophage migration
positive regulation of macrophage differentiation
interleukin-1 receptor activity
Fold enrichment
f
Weight
GFAPWtIL-1R1fl/fl
GFAPCreIL-1R1fl/fl
Percent body weight
DSS
Days
g
GFAPCreIL-1R1fl/fl
GFAPWtIL-1R1fl/fl
EPCAM
IBA1
GFAP
h
from Single Cells
Hoechst
tdTomato
tdTomato neg
tdTomato pos
i
S100b
Fold expression Rpl32 [rel. to naive tdTomatoneg]
tdTomatoneg
tdTomatopos
naive
AOM/DSS
j
SOX10CreERT2Ai14fl/fl
AOM/DSS tumor
tdTomato
IgG

Supplementary Figure 6. IL-1R activation in EGCs promotes SPP1$^+$ TAM differentiation *in vitro* and *in vivo*

a-b Murine bone marrow-derived monocytes were cultured as described in Fig. 6a. Expression levels of *Arg1, Spp1* and *C1qa*, normalized to *Rpl32*, in monocytes cultured with supernatant of TME-EGCs (*n = 7*) (**a**) or H-EGCs (*n = 4*) (**b**). **c** Representative image of EGC reporter line GFAP^{Cre}Ai14$^{fl/fl}$ showing tdTomato (magenta), GFAP (green), and SOX10 (blue) in a colon muscularis whole mount (scale bar 50 µm, *n = 3*). **d-e** Wildtype mice were subjected to the AOM/DSS model (Fig. 7a). Naive and tumor tissues were collected at d70 and processed for 3'bulk mRNA-sequencing (*n = 7* AOM/DSS, *n = 4* naive). Heatmap of selected genes upregulated in AOM/DSS compared to naive samples (**d**). Gene set enrichment analysis for differentially expressed genes in AOM/DSS-treated compared to naive mice (**e**). **f-g** GFAP^{Wt}IL-1R1$^{fl/fl}$ and GFAP^{Cre}IL-1R1$^{fl/fl}$ littermates were subjected to the AOM/DSS model (Fig. 7a). Weight curve of AOM/DSS-treated GFAP^{Wt}IL-1R1$^{fl/fl}$ (*n = 29* d7- 43, *n = 18* d44-70) and GFAP^{Cre}IL-1R1$^{fl/fl}$ mice (*n = 25* d7- 43, *n = 16* d44-70) (**f**). Representative immunofluorescence stainings of EPCAM (white), IBA1 (green) and GFAP (magenta) in tumor sections at d70 (scale bar 100 µm) (**g**). **h-i** GFAP^{Cre}Ai14$^{fl/fl}$ mice underwent the AOM/DSS model (Fig. 7a) using 1% DSS or were kept under naive conditions. Tumor or naive colon cells were isolated and FACS-sorted. Gating strategy of FACS sorting tdTomato^{pos} and tdTomato^{neg} cells (**h**). *S100b* expression levels of sorted tdTomato^{pos} glial cells versus remaining tdTomato^{neg} cells of naive and AOM/DSS-treated GFAP^{Cre}Ai14$^{fl/fl}$ mice. Expression displayed as fold to *Rpl32* and relative to naive tdTomato^{neg} cells (*n = 4*) (**i**). **j** EGC reporter SOX10^{CreERT2}Ai14$^{fl/fl}$ mice were subjected to the AOM/DSS model as described in Fig. 7a using 2% DSS. Representative image of tdTomato (magenta), IgG (green), and DAPI (blue) in tumor section at d70 (scale bar 100 µm, *n = 4*). Data are represented as mean ± SEM (i). Statistical analysis: paired t-test (a-b) and two-way ANOVA with correction for multiple comparisons (i)**.** *p < 0.05, **p < 0.005 ns not significant. Source data are provided as a Source Data file.

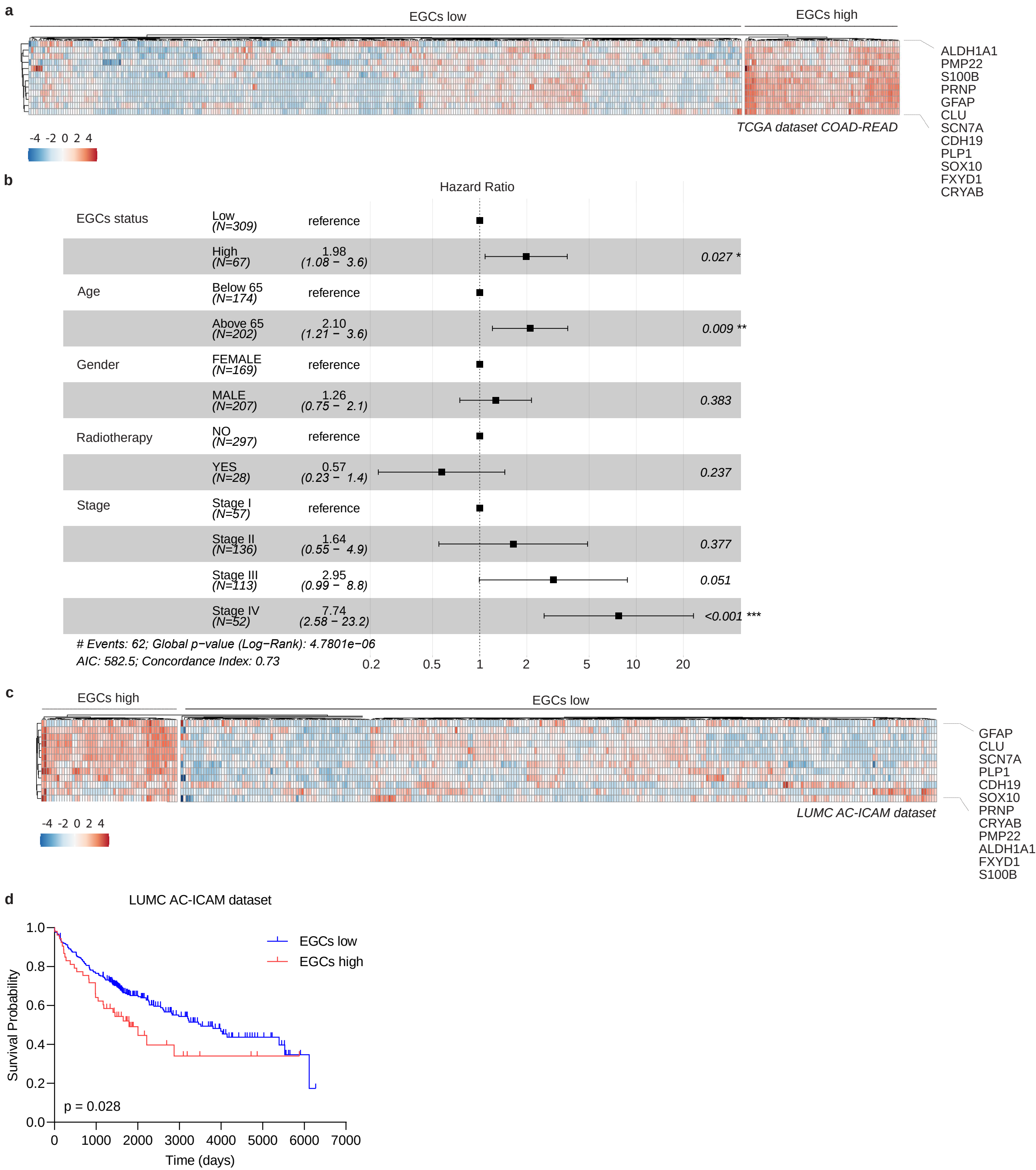

a
EGCs low
EGCs high
ALDH1A1
PMP22
S100B
PRNP
GFAP
CLU
SCN7A
CDH19
PLP1
SOX10
FXYD1
CRYAB
TCGA dataset COAD-READ
-4 -2 0 2 4
b
Hazard Ratio
EGCs status
Low (N=309) reference
High (N=67) 1.98 (1.08 − 3.6) 0.027 *
Age
Below 65 (N=174) reference
Above 65 (N=202) 2.10 (1.21 − 3.6) 0.009 **
Gender
FEMALE (N=169) reference
MALE (N=207) 1.26 (0.75 − 2.1) 0.383
Radiotherapy
NO (N=297) reference
YES (N=28) 0.57 (0.23 − 1.4) 0.237
Stage
Stage I (N=57) reference
Stage II (N=136) 1.64 (0.55 − 4.9) 0.377
Stage III (N=113) 2.95 (0.99 − 8.8) 0.051
Stage IV (N=52) 7.74 (2.58 − 23.2) <0.001 ***
Events: 62; Global p−value (Log−Rank): 4.7801e−06
AIC: 582.5; Concordance Index: 0.73
0.2 0.5 1 2 5 10 20
c
EGCs high
EGCs low
GFAP
CLU
SCN7A
PLP1
CDH19
SOX10
PRNP
CRYAB
PMP22
ALDH1A1
FXYD1
S100B
LUMC AC-ICAM dataset
-4 -2 0 2 4
d
LUMC AC-ICAM dataset
EGCs low
EGCs high
Survival Probability
1.0
0.8
0.6
0.4
0.2
0.0
p = 0.028
0 1000 2000 3000 4000 5000 6000 7000
Time (days)

Supplementary Figure 7. Tumor EGCs abundancy correlates with worse survival in patients with CRC
a-b TCGA COAD and READ patients stratified based on their expression of the EGCs signature genes (*n = 309* EGCs low, *n = 67* EGCs high). Heatmap of patients clustering (**a**). Cox logistic regression multivariate analysis of overall survival in CRC patients according to expression of EGCs signature genes and all the other relevant clinical parameters. For each variable, the reference level is the first one, P values indicate association with prognosis in this multivariate model. Error bars represent the 95% confidence interval (**b**). **c-d** LUMC AC-ICAM derived CRC patients stratified based on their expression of the EGCs signature genes (*n = 295* EGCs low, *n = 53* EGCs high). Heatmap of patients clustering (**c**) and Kaplan-Meier overall survival curve for EGCs high and low patients (**d**). Statistical analysis: Mantel cox test (d). Source data are provided as a Source Data file.

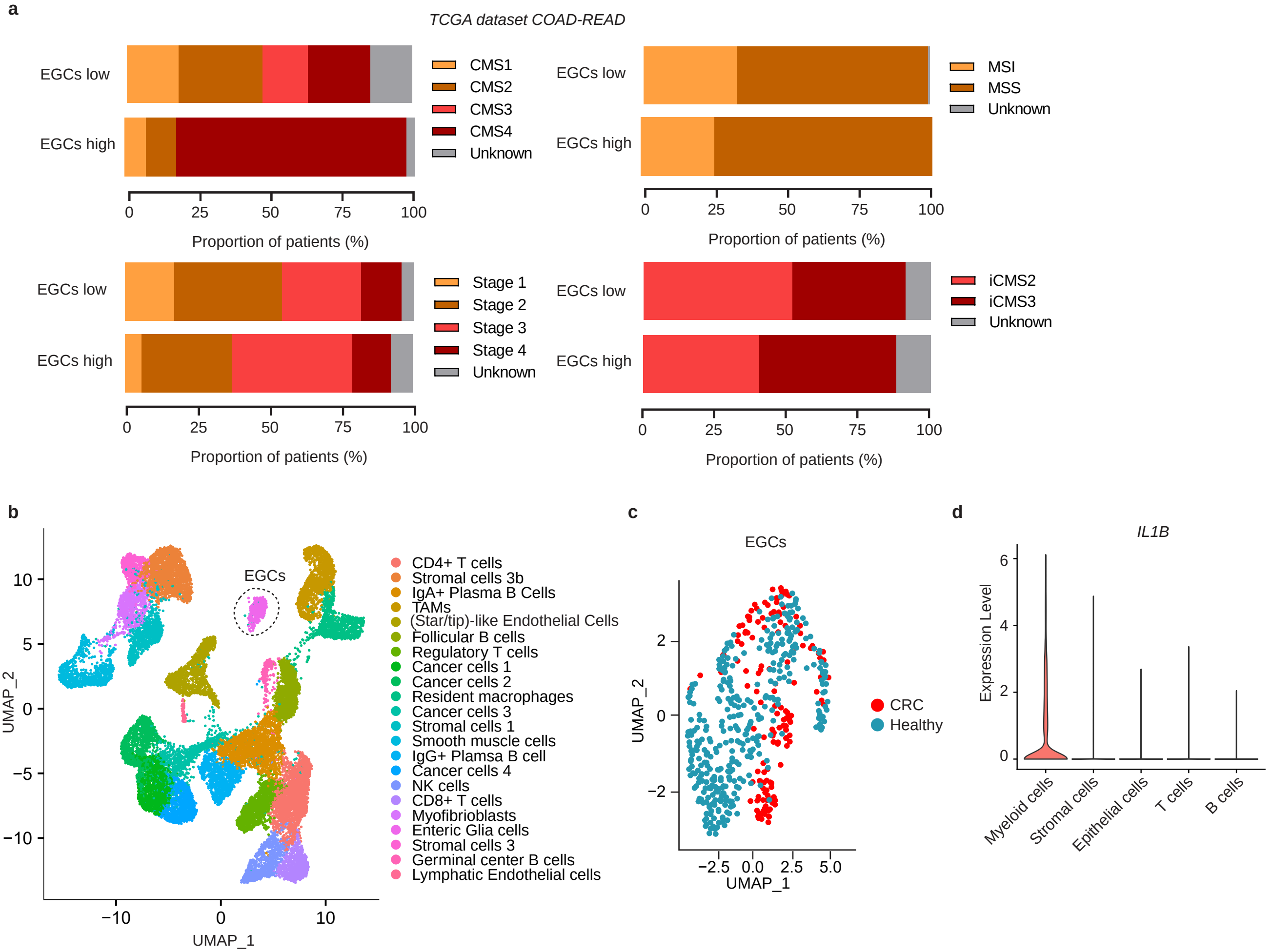

a
TCGA dataset COAD-READ
EGCs low
EGCs high
CMS1
CMS2
CMS3
CMS4
Unknown
MSI
MSS
Unknown
Proportion of patients (%)
Stage 1
Stage 2
Stage 3
Stage 4
Unknown
iCMS2
iCMS3
Unknown
b
EGCs
UMAP_1
UMAP_2
CD4+ T cells
Stromal cells 3b
IgA+ Plasma B Cells
TAMs
(Star/tip)-like Endothelial Cells
Follicular B cells
Regulatory T cells
Cancer cells 1
Cancer cells 2
Resident macrophages
Cancer cells 3
Stromal cells 1
Smooth muscle cells
IgG+ Plamsa B cell
Cancer cells 4
NK cells
CD8+ T cells
Myofibroblasts
Enteric Glia cells
Stromal cells 3
Germinal center B cells
Lymphatic Endothelial cells
c
EGCs
CRC
Healthy
UMAP_1
UMAP_2
d
IL1B
Expression Level
Myeloid cells
Stromal cells
Epithelial cells
T cells
B cells

Supplementary Figure 8. EGCs are enriched in CRC tumors of patients with CMS4

a TCGA COAD and READ patients stratified based on their expression of the EGCs signature genes (*n = 309* EGCs low, *n = 67* EGCs high). The proportion of the EGCs high and low patients classified in the different disease stages (top left), MSI/MSS (top right) CMS subtypes (bottom left) and iCMS subtypes (bottom right). **b-d** Transcriptome analysis of human CRC and healthy colon tissues in the KUL3 Dataset, Lee H. O. et al.[1] (*n = 5*). UMAP of full scRNA-seq dataset, indicating the EGCs cluster (**b**). UMAP of isolated EGCs cluster (**c**). Violin plot showing expression of *IL1B* in the tumor microenvironment clusters (**d**). Source data are provided as a Source Data file.

REFERENCES

1. Lee, H. O. *et al.* Lineage-dependent gene expression programs influence the immune landscape of colorectal cancer. *Nat. Genet.* **52**, 594–603 (2020).

2. Drokhlyansky, E. *et al.* The Human and Mouse Enteric Nervous System at Single-Cell Resolution. *Cell* **182**, 1606–1622 (2020).

3. Kinchen, J. *et al.* Structural Remodeling of the Human Colonic Mesenchyme in Inflammatory Bowel Disease. *Cell* **175**, 372–386 (2018).

4. Zhang, L. *et al.* Single-Cell Analyses Inform Mechanisms of Myeloid-Targeted Therapies in Colon Cancer. *Cell* **181**, 442–459 (2020).
