# Supplementary material for: IL-1R signaling drives enteric glia-macrophage interactions in colorectal cancer
Source: Nat Commun. 2024 Jul 19;15:6079. doi: 10.1038/s41467-024-50438-2 (PMC11271635; doi:10.1038/s41467-024-50438-2)
Supplement: Supplementary file 3 — Description of Additional Supplementary Files [file 41467_2024_50438_MOESM3_ESM.pdf]

**Description of Additional Supplementary Files**

Supplementary Data 1. Differentially expressed genes and GO terms in Modules of weighted gene correlation network analysis (WGCNA), related to Figure 2C.

Supplementary Data 2. Differentially expressed genes in EGCs transcriptomic analysis and significantly increased proteins in IL-1 stimulated EGCs mass spectrometry analysis. Related to Figures 2, 4 and 8.

Supplementary Data 3. Differentially expressed genes between full-thickness AOM/DSS-treated colonic tumors and naive colonic tissues identified by 3'bulk mRNA-seq. Related to Supplementary Figure 6.
