# Supplementary material for: IL-1R signaling drives enteric glia-macrophage interactions in colorectal cancer
Source: Nat Commun. 2024 Jul 19;15:6079. doi: 10.1038/s41467-024-50438-2 (PMC11271635; doi:10.1038/s41467-024-50438-2)
Supplement: Supplementary file 7 — Reporting Summary [file 41467_2024_50438_MOESM7_ESM.pdf]

nature portfolio

Corresponding author(s): Gianluca Matteoli

Last updated by author(s): Jun 24, 2024

Reporting Summary

Nature Portfolio wishes to improve the reproducibility of the work that we publish. This form provides structure for consistency and transparency in reporting. For further information on Nature Portfolio policies, see our Editorial Policies and the Editorial Policy Checklist.

Statistics

For all statistical analyses, confirm that the following items are present in the figure legend, table legend, main text, or Methods section.

n/a	Confirmed	
☐	☒	The exact sample size (n) for each experimental group/condition, given as a discrete number and unit of measurement
☒	☐	A statement on whether measurements were taken from distinct samples or whether the same sample was measured repeatedly
☐	☒	The statistical test(s) used AND whether they are one- or two-sided<br>*Only common tests should be described solely by name; describe more complex techniques in the Methods section.*
☒	☐	A description of all covariates tested
☐	☒	A description of any assumptions or corrections, such as tests of normality and adjustment for multiple comparisons
☐	☒	A full description of the statistical parameters including central tendency (e.g. means) or other basic estimates (e.g. regression coefficient) AND variation (e.g. standard deviation) or associated estimates of uncertainty (e.g. confidence intervals)
☐	☒	For null hypothesis testing, the test statistic (e.g. F, t, r) with confidence intervals, effect sizes, degrees of freedom and P value noted<br>*Give P values as exact values whenever suitable.*
☒	☐	For Bayesian analysis, information on the choice of priors and Markov chain Monte Carlo settings
☒	☐	For hierarchical and complex designs, identification of the appropriate level for tests and full reporting of outcomes
☐	☒	Estimates of effect sizes (e.g. Cohen's d, Pearson's r), indicating how they were calculated

Our web collection on statistics for biologists contains articles on many of the points above.

Software and code

Policy information about availability of computer code

Data collection	All data analyzed within this manuscript are publicly available. No additional software was used for the data collection process
Data analysis	No custom algorithms were used in the analysis. Code for any specific analysis is available from authors upon request.<br>1) Liquid Chromatography/Mass Spectrometry (LC/MS): Peptide identification was done with an in-house Mascot server version 2.8.1. Mascot results were evaluated by the Percolator algorithm version 3.02.12. The statistical analyses of the peptide-spectrum match (PSM) level data were done by the Core Unit for Bioinformatics Data Analysis of the University of Bonn. Non-unique peptides and single-hit proteins (proteins identified/quantified by only one peptide) were filtered-out prior to the statistical analysis. From all available fractions, only those with the least number of missing values per feature and maximum average intensity across all TMT labels were selected. The PSM-level data were then log-transformed and scaled such that all the samples have the same median values (median normalization method). Next, the normalized data was aggregated to protein-level by applying the Tukey's median polish method. Analyses were carried out in R environment (R version 4.2) using an in-house developed workflow. The statistical analysis was performed using the R package limma (Ritchie et al., 2015).<br>2) Bulk RNA sequencing: Quality control of raw reads was performed with FastQC v0.11.7. Adapters were filtered with ea-utils fastq-mcf v1.05. Resulting BAM alignment files were handled with Samtools v1.5. Quantification of reads per gene was performed with HT-seq Count v0.10.0, Python v2.7.14. Count-based differential expression analysis was done with Bioconductor package DESeq2. Data visualization was prepared using ggplot2 R package (v3.4.1) or pheatmap (v1.0.12). Further analysis was performed with: R package WGCNA (v1.72.1)<br>OR Data were analyzed using PartekFlow software (V10.0.23.0720) available from https://www.partek.com/partek-flow/#features with the Lexogen12112017 pipeline and Ensemble transcripts release 102 for mm10 mouse alignment. Briefly, two adapter-trimming steps and one base-trimming step were performed before alignment was done by star2.5.3a. Post-alignment QC was performed and reads were quantified to the annotation model. Gene counts were normalized and data were further analyzed by gene-specific analysis. Visualization was done with PartekFlow software (V10.0.23.0720) and GraphPad Prism 6 (V6.07).<br>To quantify the transcripts per million (TPM) values from the bulk RNA-seq data of adult derived EGCs (previously published in Schneider et

al., 2021 and Schneider et al. 2022), PartekFlow software (V10.0.23.0720) available from https://www.partek.com/partek-flow/#features. The TPM values for bulk RNA-seq of embryonic neurosphere-derived EGCs were obtained by processing fastq files using nf-core/rnaseq pipeline (nf-co.re/rnaseq/3.12.0) where GRCh37 was used as reference genome and Salmon was used for quantification (Ewels et al. 2020).
3) Single cell RNA sequencing: Seurat R package (v3.1.3)., 'ClusterProfiler' package (v4.6.0), NicheNet (nichenetr R package; v1.1.0), (scGSVA) R package (v0.0.11).
4) TCGA and LUMC AC-ICAM dataset analysis: The processed gene expression RNA-seq data of COADREAD was downloaded using the UCSCXenaTools (ref: https://joss.theoj.org/papers/10.21105/joss.01627) R library. Further analysis was performed with R package CMSClassifier (v1.0.0), survival (v3.5.5) and survminer (v0.4.9)

For manuscripts utilizing custom algorithms or software that are central to the research but not yet described in published literature, software must be made available to editors and reviewers. We strongly encourage code deposition in a community repository (e.g. GitHub). See the Nature Portfolio guidelines for submitting code & software for further information.

Data

Policy information about availability of data

All manuscripts must include a data availability statement. This statement should provide the following information, where applicable:

- Accession codes, unique identifiers, or web links for publicly available datasets
- A description of any restrictions on data availability
- For clinical datasets or third party data, please ensure that the statement adheres to our policy

The scRNA-seq (related to Fig. 3 and Supplementary Fig. 4) and bulk RNA-seq (related to Fig. 2 and Supplementary Fig. 3 and 5b) data generated for this study are deposited in the Gene Expression Omnibus (GEO) database under the GEO accession code GSE231804. The bulk RNA-seq (related to Supplementary Fig. 6), data generated for this study are deposited in the Gene Expression Omnibus (GEO) database under the GEO accession code GSE231709. The mass spectrometry proteomics data generated for this study (related to Fig. 4) have been deposited to the ProteomeXchange Consortium via the PRIDE partner repository with the dataset identifier PXD045911. Bulk RNA-seq data (related to Supplementary Fig 5b) were published previously (PMID: 33332729, PMID: 35962064) and can be accessed via GSE205610 and GSE134943. The remaining data are available within the article, supplementary information or source data file.

Research involving human participants, their data, or biological material

Policy information about studies with human participants or human data. See also policy information about sex, gender (identity/presentation), and sexual orientation and race, ethnicity and racism.

Reporting on sex and gender	N/a
Reporting on race, ethnicity, or other socially relevant groupings	N/a
Population characteristics	The covariate details of the patient (collected at the UZ/KU Leuven biobank according to protocol S66460) used in the IF staining in Figure 8a are as follows:<br>Age: 68 years old,<br>Gender: Female,<br>Pathologic subtype: Moderately Differentiated adenocarcinoma, MSS/MSI status: MSS,<br>Site: ascending Colon,<br>Sidedness: Right,<br>Stage: 2 and T3N0M0,<br>iCMS status: iCMS3<br><br>The covariate-relevant characteristics for the TCGA COAD and READ data, used in the analysis for Figure 8b-c and Supplementary Figures 7 and 8a, are detailed in Supplementary Figure 7b. More information on the population characteristics can be found in The Cancer Genome Atlas Program.<br><br>All covariate-relevant characteristics for the KUL3 data, which were used to generate Figure 8d-f and Supplementary Figures 8b-d, can be found in the original publication by Lee et al., 2020, in Nature Genetics.
Recruitment	N/a
Ethics oversight	formalin-fixed paraffin-embedded human CRC samples were used from biobank at the UZ/KU Leuven according to protocol S66460

Note that full information on the approval of the study protocol must also be provided in the manuscript.

Field-specific reporting

Please select the one below that is the best fit for your research. If you are not sure, read the appropriate sections before making your selection.

[x] Life sciences [ ] Behavioural & social sciences [ ] Ecological, evolutionary & environmental sciences

For a reference copy of the document with all sections, see nature.com/documents/nr-reporting-summary-flat.pdf

Life sciences study design

All studies must disclose on these points even when the disclosure is negative.

Sample size	sample size calculations was performed a priori, compute required sample size - given α = 5%, power = 80% and effect size (dependent on test experiment)
Data exclusions	Outliers were excluded by 'ROUT method' using GraphPad prism
Replication	All in vitro experiments were repeated at least three times. All in vivo experiments at least two times. All attempts at replication were successful.
Randomization	Mice were randomly assigned into treatment groups.
Blinding	Not applicable - study is not a clinical trial

Reporting for specific materials, systems and methods

We require information from authors about some types of materials, experimental systems and methods used in many studies. Here, indicate whether each material, system or method listed is relevant to your study. If you are not sure if a list item applies to your research, read the appropriate section before selecting a response.

Materials & experimental systems

n/a	Involved in the study	
☐	☒	Antibodies
☐	☒	Eukaryotic cell lines
☒	☐	Palaeontology and archaeology
☐	☒	Animals and other organisms
☒	☐	Clinical data
☒	☐	Dual use research of concern
☒	☐	Plants

Methods

n/a	Involved in the study	
☒	☐	ChIP-seq
☐	☒	Flow cytometry
☒	☐	MRI-based neuroimaging

Antibodies

Antibodies used

Immunofluorescence:
Antibody Catalog number Clone number Dilution
Chicken IBA1 234009 (synaptic systems) Ch311H9, 1:400
Rat CD326 (EPCAM) 118201 (Biolegend) G8.8, 1:1000
Rabbit KI67 ab16667 (abcam) SP6, 1:400
Rabbit IBA1 ab178847 (abcam) EPR16589, 1:400
Rabbit IL-6 ab7737 (abcam) polyclonal, 1:100
Chicken GFAP 829401 (Biolegend) Poly28294, 1:1000
rat anti-F4/80, BioRad, MCA497, Cl:A3-1, 1:500
rabbit anti-GFAP Dako, GA524, Polyclonal, 1:300
DAPI (Sigma-Aldrich) D9542, 1:1000
mouse anti-pan cytokeratin (Invitrogen, ThermoFisher Scientific) # MA5-12231, C-11, 1:500
rabbit anti-S100B (Dako), GA504, Polyclonal, 1:500
mouse anti-CD68 (Invitrogen, ThermoFisher Scientific) # 14-0688-82, KP1, 1:100
Rabbit IgG: 011-000-003 (Dianova), 1:2000
Hoechst: 33258, (Invitrogen, ThermoFisher Scientific) H3569, 1:1000
goat anti-SOX10 (R&D Systems) AF2864, polyclonal, 1:300
Donkey anti-chicken AF568 A78950 (Invitrogen, Thermo Fisher Scientific) polyclonal, 1:800
Donkey anti-rat AF488 A-21208 (Invitrogen, Thermo Fisher Scientific) polyclonal, 1:800
Donkey anti-rabbit AF647 6444-31 (SouthernBiotech) polyclonal, 1:800
Donkey anti-rabbit FITC 711-095-152 (Dianova) polyclonal, 1:800
Donkey anti-chicken CF633 SAB4600127 (Sigma Aldrich) polyclonal, 1:800
Donkey anti-chicken FITC 703-095-155 (Jackson ImmunoResearch) polyclonal, 1:800
Donkey anti-goat CF647 SAB4600175 (Sigma) polyclonal, 1:800
Alexa fluor 647 donkey anti-rabbit (Invitrogen, ThermoFisher Scientific) # A-31573,polyclonal, 1:800
Alexa Fluor 488 goat anti-mouse (Invitrogen, ThermoFisher Scientific) # A-11001, polyclonal, 1:800
donkey anti-rabbit Cy5 (Jackson) AB_2340607, polyclonal, 1:400
donkey anti-goat AF488 (Invitrogen, ThermoFisher Scientific) # A-11055, polyclonal, 1:500
goat anti-SOX10 custom-made (aliquot kindly provided by Prof. Wegner, University of Erlangen), 1:1000

Western Blot:
mouse anti-GFAP antibody (Cell Signaling), #3670,GA5, 1:500
mouse anti-vinculin antibody (Sigma-Aldrich), V9131, hVIN-1, 1:5000
anti-mouse antibody conjugated to horseradish peroxidase (Dako Agilent Technologies), P0447, Polyclonal, 1:5000

FACS:
Anti Conjugate Company Cat. No. Clone Dilution
ARG1 Pe-Cy7 eBioscience 25-3697-82 A1efF5, 1:100
C1Q FITC Tebubio 7501F RmC7H8, 1:50
CD11b BUV395 BD Horizon 563553 M1/70, 1:1000
CD11b PE-Cy7 BD Pharmingen 552850 M1/70, 1:400
CD19 PE-Cy5 eBioscience 15-0193-82 eBio1D3, 1:400
CD3 Alexa Fluor 700 BioLegend 100215 17A2, 1:200
CD31 BV421 BioLegend 102423 390, 1:100
CD326 PE-Cy7 BioLegend 118215 G8.8, 1:200
CD3e eFluor 450 eBioscience 48-0032-82 17A2, 1:100
CD4 BV605 BioLegend 100548 RM4-5, 1:400
CD44 AF700 BioLegend 103026 IM7, 1:200
CD45 BUV805 BD OptiBuild 748370 30-F11, 1:1000
CD45 APC-eFluor 780 eBioscience 47-0451-82 30-F11, 1:250
CD64 BV711 BioLegend 139311 X54-5/7.1, 1:200
CD8a APC-Cy7 eBioscience 25-5773-82 FJK-16s, 1:200
FOXP3 PE-Cy7 eBioscience 12-5773-82 FJK16S, 1:50
GP38 (PDPN) Alexa Fluor 488 BioLegend 127405 8.1.1, 1:100
IL-1α PE BioLegend 503203 ALF-161, 1:50
IL-1β (Pro-form) APC eBioscience 17-7114-80 NJTEN3, 1:50
Live Dead eFluor 506 eBioscience 65-0866-14, 1:400
Live Dead 7-AAD BD Pharmingen 51-68981E, 1:100
Ly6C BV421 BioLegend 128043 HK1.4, 1:500
Ly6C BV650 BioLegend 128049 HK1.4, 1:200
Ly6C FITC BD Pharmingen 553104 AL-21, 1:300
Ly6G BUV563 BD Horizon 612921 IA8, 1:250
Ly6G APC BD Pharmingen 560599 1A8, 1:300
MHCII APC-eFluor 780 eBioscience 47-5321-82 M5/114.15.2, 1:400
MHCII BV510 BioLegend 107636 M5/114.15.2, 1:500
SiglecF eFluor 660 eBioscience 50-1702-80 1RNM44N, 1:200
SPP1 PE R&D systems IC808P, 1:25

Validation

All antibodies were used and validated in previous publications.
In regard to the self-made goat anti-SOX10, it was kindly provided by Prof. Wegner, University of Erlangen and validated in PMID: 32672815.

Eukaryotic cell lines

Policy information about cell lines and Sex and Gender in Research

Cell line source(s)

Murine colon adenocarcinoma cell line MC38 (NCI, ENH204-FP) was kindly provided by Prof. Max Mazzone (VIB - KU Leuven).
AKPT tumor-derived cells were kindly provided by Prof. Owen J. Sansom (Cancer Research UK Beatson Institute).

Authentication

The MC38 and AKPT cell line was not authenticated

Mycoplasma contamination

The MC38 and AKPT cell line was tested negative for mycoplasma

Commonly misidentified lines
(See ICLAC register)

No commonly misidentified cell lines were used in the study.

Animals and other research organisms

Policy information about studies involving animals; ARRIVE guidelines recommended for reporting animal research, and Sex and Gender in Research

Laboratory animals

WT C57BL/6J (JAX:000664), CCR2-/- (JAX: 004999), IL-1R1-/- (JAX:003245), PLP1CreERT2iDTR (JAX:005975 and JAX:007900), PLP1CreERT2Ai14fl/fl (JAX:005975 and JAX:007908), GFAPCreIL-1R1fl/fl (JAX:012886 and JAX:028398), GFAPCreAi14fl/fl (JAX:012886 and JAX:007908), Sox10CreERT2Ai14fl/fl [Sox10CreERT2 (kindly provided by Dr. Vassilis Pachnis (Laranjeira et al., 2011), (Ai14fl/fl JAX:007908)], and SOXCreERT2IL-6fl/fl (SOX10CreERT2 as mentioned previously and IL-6fl/fl were kindly provided by Juan Hidalgo's lab (Quintana, A. et al., 2013)).
Male and female, age 8-12 weeks at start of the experiment.

Wild animals

Study did not involved wild animals

Reporting on sex	No sex based analysis were performed. When relevant, information on sex were indicated in the Material and Methods section.
Field-collected samples	Study did not involved field-collected samples
Ethics oversight	All experimental procedures were approved by the Animal Care and Animal Experiments Ethical Committee of KU Leuven (208/2018, 159/2021 and 213/2018) or by the Regional Office for Nature, Environment and Consumer Protection of North-Rhine-Westphalia, Germany (81-02.04.2021.A424).

Note that full information on the approval of the study protocol must also be provided in the manuscript.

Flow Cytometry

Plots

Confirm that:

- [x] The axis labels state the marker and fluorochrome used (e.g. CD4-FITC).
- [x] The axis scales are clearly visible. Include numbers along axes only for bottom left plot of group (a 'group' is an analysis of identical markers).
- [x] All plots are contour plots with outliers or pseudocolor plots.
- [x] A numerical value for number of cells or percentage (with statistics) is provided.

Methodology

Sample preparation	Tumor-bearing mice were sacrificed at the described time points. After peeling off the muscularis layer from the orthotopic tumors, tissues were first cut in 1 mm pieces, and then went under mechanical and enzymatic digestion for 30 min in DMEM with 2.5% FBS, 100 µg/mL Penicillin and Streptomycin, 200 U/mL collagenase IV (Gibco, ThermoFisher Scientific) and 125 µg/mL type II dispase (Gibco, ThermoFisher Scientific). AOM/DSS induced tumors and healthy colon samples were peeled off the muscularis layer and underwent epithelial removal by vigorous shaking in Hanks' balanced salt solution (HBSS) with phenol red (Gibco, ThermoFisher Scientific) containing 1% FBS, 100 µg/mL Penicillin and Streptomycin, 1 mM EDTA (Invitrogen, ThermoFisher Scientific) and 1 mM dithiothreitol (DTT) (Sigma- Aldrich) for 8 min at 37°C. A second incubation step was performed for 8 min at 37°C in the same medium without DTT. After washing in wash medium (DMEM with 2.5% FBS and 100 µg/mL Penicillin and Streptomycin), the remaining tissue was cut into small pieces and digested for 30 min at 37°C in pre-warmed alpha Minimum Essential Medium (MEM) (Lonza) containing 5% FBS, 100 µg/mL Penicillin and Streptomycin, 5 U/mL DNase (Roche), 1 mg/mL dispase (Gibco, ThermoFisher Scientific), 1.25 mg/mL Collagenase D (Roche) and 0.85 mg/mL Collagenase V (Sigma-Aldrich). Independent of tumor origin, cells were then filtered through a 70-µm cell strainer (BD Falcon), washed with PBS, and stained with fluorophore-conjugated antibodies.
Instrument	BD Symphony A5 Cell Analyzer (BD Biosciences), Sony MA9000 sorter and BD FACSAria III cell sorter.
Software	FlowJo v.10.6.1.
Cell population abundance	The abundance of FACS-sorted cells was determined by Sony MA9000 sorter software or BD FACSDiva 9.0.1 software, and >98% purity was confirmed
Gating strategy	For all experiments, population of interest were gated based on size and granularity in the initial FSC-A and SSC-A gate. Doublet exclusion was performed by eliminating non-linear ratios of FSC-A and FSC-H in the subsequent gate. Dead cells were excluded based on viability dye stain. Further gates were set as indicated in the supplementary data. Fluorescent-minus one (FMO) controls were used to determine the cut-off points between negative and positive populations.

- [x] Tick this box to confirm that a figure exemplifying the gating strategy is provided in the Supplementary Information.
